# Numerical Simulation of a Novel Sensing Approach Based on Abnormal Blocking by Periodic Grating Strips near the Silicon Wire Waveguide

**DOI:** 10.3390/s18061707

**Published:** 2018-05-25

**Authors:** Andrei Tsarev, Eugeny Kolosovsky, Francesco De Leonardis, Vittorio M. N. Passaro

**Affiliations:** 1 Laboratory of Optical Materials and Structures, Rzhanov Institute of Semiconductor Physics, SB RAS, 630090 Novosibirsk, Russia; tsarev@isp.nsc.ru (A.T.); kolos@isp.nsc.ru (E.K.); 2 Laboratory of Semiconductor and Dielectric Materials, Physics Department, Novosibirsk State University, 630090 Novosibirsk, Russia; 3 Photonics Research Group, Dipartimento di Ingegneria Elettrica e dell’Informazione, Politecnico di Bari, Via E. Orabona 4, 70125 Bari, Italy; francesco.deleonardis@poliba.it

**Keywords:** diffraction, segmented gratings, silicon and compounds, optical sensors

## Abstract

This paper discusses the physical nature and the numerical modeling of a novel approach of periodic structures for applications as photonic sensors. The sensing is based on the high sensitivity to the cover index change of the notch wavelength. This sensitivity is due to the effect of abnormal blocking of the guided wave propagating along the silicon wire with periodic strips overhead it through the silica buffer. The structure sensing is numerically modeled by 2D and 3D finite difference time domain (FDTD) method, taking into account the waveguide dispersion. The modeling of the long structures (more than 1000 strips) is accomplished by the 2D method of lines (MoL) with a maximal implementation of the analytical feature of the method. It is proved that the effect of abnormal blocking could be used for the construction of novel types of optical sensors.

## 1. Introduction

Grating assisted couplers [1,2,3,4,5,6,7] belong to the most popular optical devices utilizing periodic structures. The grating in the vicinity of the single mode waveguides is usually used to couple the external optical beam to the guided mode [4,5] and to construct an optical filter by interaction with the backward-reflected guided mode [6]. Very often, grating is also used to couple the guided modes in two closely spaced optical waveguides forming the optical filter [8,9,10]. In all cases, the grating periodicity provides the phase-matching between different interacting waves, according to the commonly used Bragg condition [1]:

|*β*_1_ − *β*_2_| = *K* (1)

where *β_i_ =* 2*π N_i_/λ*_0_*, N_i_*—the interacting modes effective mode index, *λ*_0_ is the optical wavelength, *K =* 2*π p*/*Λ, Λ* is the grating period, *p* = 1, 2, 3, etc.—is the diffraction order. 

**Figure 1:**
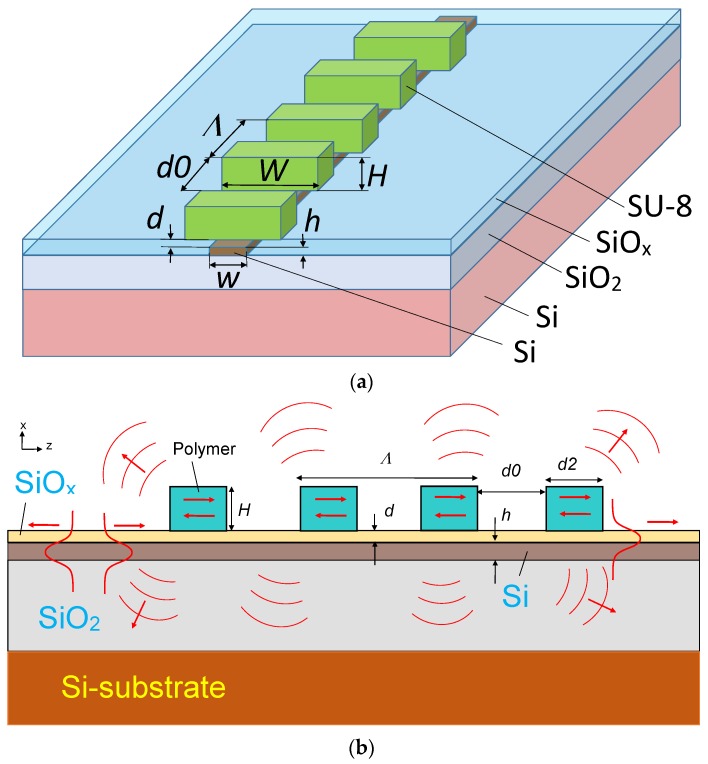
Structure design for modeling of guided wave propagation in the silicon waveguide, which is evanescently coupled to the segmented grating. The fundamental TE_0_ mode starts at Z = 0 and propagates under the periodic segmented grating formed by multiple dielectric strips. (**a**) 3D view; (**b**) 2D view.

A new outlook on the well-known grating filter design [1,2] is formulated in the present paper, but with the fully etched grating (looks like a periodic segmented structure of the dielectric strip inserts, see Figure 1) constructed near the boundary of the single mode waveguide. Similar polymer grating is previously used to design the wide aperture quasi-single mode strip and grating loaded waveguide [11] on the silicon-on-insulator (SOI) structure. This grating is coupled to the waveguide modes by evanescent fields. Typically, such gratings are used as a periodic perturbation to couple the reverse directed guided modes. Thus, this optical element could be used as a notch filter [10] for the optical wavelength satisfying the Bragg condition (1). In this case, we have the guided wave blocking in the forward direction when the launch mode is totally transmitted into the same wave of the opposite direction.

Our investigations prove [12] that the similar segmented grating (as a set of periodically spaced dielectric strips) could provide strong blocking of a guided wave without inducing any backward-directed guided wave. This unusual feature could be explained by the evanescent coupling of the guided mode with a virtual leaky mode supported by the grating area and which is further radiated from the structure. Thus, the guided wave could be blocked, but this phenomenon will be not supported by the back-reflected guided wave. This abnormal guided wave blocking effect [12] could be used for the construction of novel notch filters with negligible back-reflection as well as optical sensors.

## 2. The Effect of Abnormal Guided Wave Blocking

### 2.1. Genreral Description of the Abnormal Blocking in the Guided Wave Structure with the Segmented Grating

The structure design which illustrates the effect of abnormal blocking is shown in Figure 1. The optical wave propagates in the silicon single mode waveguide (silicon wire) on the silicon-on-insulator (SOI) structure. The segmented grating is constructed by polymer SU-8 fully etched dielectric strips with the width *W* and height *H* arranged symmetrically above the silicon waveguide having the width *w* and height *h*, and which are spaced by a thin silica buffer (*d*~100–400 nm). Manufactured technology could be similar to the case of polymer fiber to silicon wire adiabatic coupler [13]. 

In this optical element. the guided mode of the silicon waveguide is coupled to the grating by the evanescent field and this effect could be controlled by the height of the silica buffer (*d*), grating period (*Λ*), height (*H*), width (*W*) and length (*d*2) of grating segments, as well as by the number of periods (*M*) in the grating design.

The arrows in Figure 1 indicate the incident fundamental TE_0_ mode (left) and the scattered fields. Numerical simulation of such structures has been performed using the finite difference time domain (FDTD) method through the commercial software package RSoft-SYNOPSYS [14]. 

When the guided optical beam, which contains a broad spectral range, arrived in the grating area (see Figure 1), one can see different optical processes which depend on the ratio between the optical wavelength *λ*_0_ and grating period *Λ*. It can be the well-known effect of the Bragg reflection to the guided wave propagated in silicon wire in the opposite direction or the broadband interaction with radiation modes that provides the out coming optical wave with the radiation angle depending on the optical wavelength.

Our investigation is focused on the resonance-type interaction of incoming guided wave with the virtual leaky wave that is supported by the periodic grating structure, which is coupled by evanescent field with the underplayed silicon wire. This type of diffraction we call as “abnormal blocking” [12] because of its unusual feature. Namely, this interaction is a resonance type like a Bragg reflection and supported by the outcoming optical beam like a coupling with the radiated modes. But on the contrary with the last process, the coupling to radiated modes has a resonance feature and thus this effect of “abnormal blocking” can be used for sensing applications. Depending on the grating order *p*, the Bragg condition could be satisfied for the forward diffraction (*p* = 1) from guided to the virtual leaky wave, or to backward diffraction (*p* = 3) to the virtual leaky of the opposite direction. 

From the Bragg condition (1) on can derive relation between the effective indices *N_L_* and *N_g_* of the leaky and guided waves in the silicon waveguide with the segmented grating structure:

*N_L_* = ±*N_g_* − *p∙λ/Λ* (2)

The changing of the grating environment index by the amount *dN* leads to the change in the condition (2) of diffraction observation. This change is the subject of the measurement by the optical sensor. The main sensor parameters could be derived from Equation (2) in the form of the following set of equations:

*∂N_L_/∂n* = *±∂N_g_/∂n − ∂λ/∂n∙p/Λ* (3)

*S_n_* = ∂*λ/∂n* = *Λ/p*∙(*∂N_L_/∂n* ± *∂N_g_/∂n*) (4)

Equation (3) is characterized by the mode index sensitivity of the sensor. In order to improve the sensitivity, one can use the waveguide near the cutoff, the slot [15,16] or segment waveguide structures [17,18]. Equation (4) describes the homogeneous sensitivity of the typical sensor which is working by measuring the Drop wavelength of the structure. One can see that the forward diffraction (*p* = 1) provides the better (*p*-times sensitivity) then backward diffraction (*p* = 3) thus this process is the subject of our investigation.

### 2.2. Not-Vertical Etching Profile

**Figure 2:**
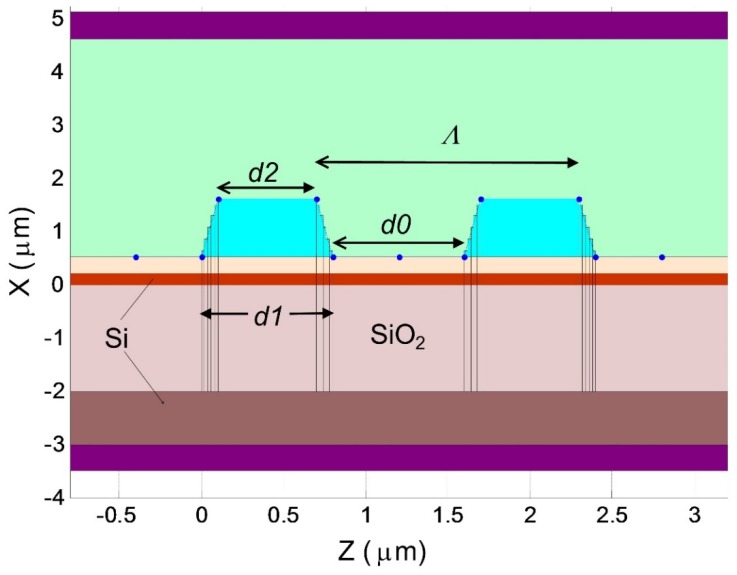
A general view of the waveguide structure with inclined boundaries for transmission of light in a photonic silicon wire (Si) containing a segmented structure of a large number of periodically arranged dielectric inserts. The inclined boundaries are determined by the parameters: *d*1, *d*2, *dD = d*1 − *d*2 and *d*0. The top and bottom rectangular layers represent the PML layered to suppress the back reflection from the boundary of the simulation region.

Firstly, the effect of abnormal blocking was examined for the case of periodical structure of SU-8 polymer with low refractive index (1.56) and vertical walls [12]. Calculations show that the effect of the anomalous blocking is also observed for the more general case of segmental structures made of materials with different refractive indices and a not-vertical etching profile (see Figure 2). Our further investigation shows that the different optical index contrast and slope of grating strip boundaries modify the spectral characteristics of the abnormal blocking, but without affecting its basic properties. 

**Figure 3:**
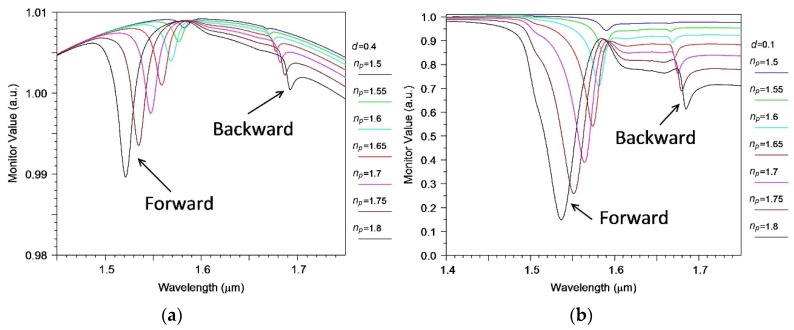
Transmission spectrum of a silicon waveguide in the presence of a periodic segmented structure made by a material with different refractive index surrounded by water for two thicknesses d of the buffer oxide layer between the waveguide and the segmental structure. (**a**) *d* = 0.4 µm; (**b**) *d* = 0.1 µm. The grating period is 1.3 µm.

In particular, the transmission spectrum of a silicon waveguide in the presence of a periodic segmented structure made of a material with different refractive indices in the water environment is presented in Figure 3 (usually used for analysis as optical sensors). It is seen that the refractive index change leads to a change in amplitude and position of the minimum of the signal transmission, but not any qualitative changes. For the case of a thick (0.4 µm) buffer oxide layer, a relatively small amount of depletion (2%) is observed for a short structure of 128 segments. In order to increase the damping of the transmission signal, it is needed to increase the number of segments, and/or use a thinner buffer layer, for example, 0.1 µm thick (see Figure 3b). 

**Figure 4:**
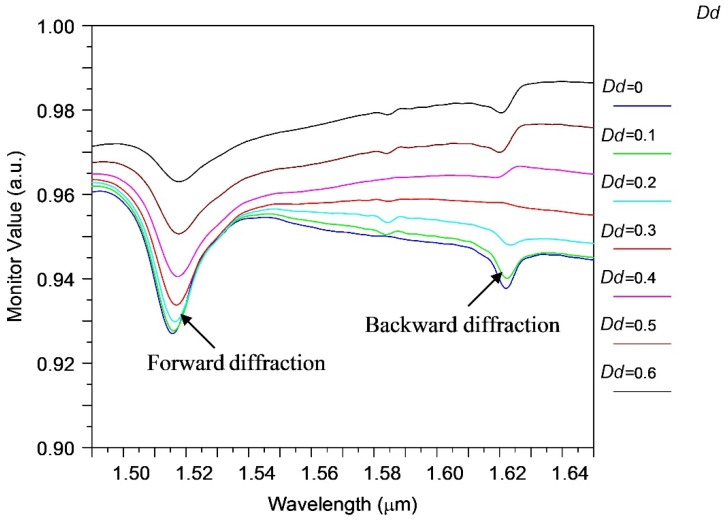
Transmission spectrum of silicon waveguide in the presence of periodic segmental structure surrounded by water for different inclination angles determined by *Dd*.

During the manufacturing of such structures, sloping boundaries could be formed (see Figure 2), whereby the width of the segment at the top (*d*2) is less to the width of its base (*d*1*)* by the amount of *Dd = d*1 − *d*2. Calculations show that with increasing the inclination angle of the segment boundaries (depending on *Dd*), a decrease in the efficiency of the abnormal blocking occurs, but it does not affect the qualitative characteristics of this effect (see Figure 4). This is very important in practice, because real structures can have inclined boundaries as a result of selective etching methods.

### 2.3. Effective Index Method and Implementation of 2D FDTD

The optical properties of segmented waveguides can be efficiently calculated by 3D FDTD method by analyzing the propagation of a short pulse through its structure and constructing a Fourier spectrum of the transmitted signal [14]. However, due to the huge demand on numerical resources for such modeling (very memory and time consuming), this method can be used only for relatively small structures (no more than 64–128 segments). In many practical cases, especially when analyzing notch filters and optical sensors, the use of a longer structure is required, where it is convenient to apply the effective index method (EIM) [19].

Previously, we have shown that the EIM method has a fundamental limitation [20,21], which is revealed when trying to analyze the pulsed excitation of waveguide structures by 2D FDTD method. It takes place due to the fact that the two-dimensional EIM approximation does not allow to take into account any waveguide dispersion, which is the fundamental feature in the propagation of a short pulse containing a wide spectral composition. Therefore, the classical EIM can be used only for the monochromatic excitation of a waveguide.

**Figure 5:**
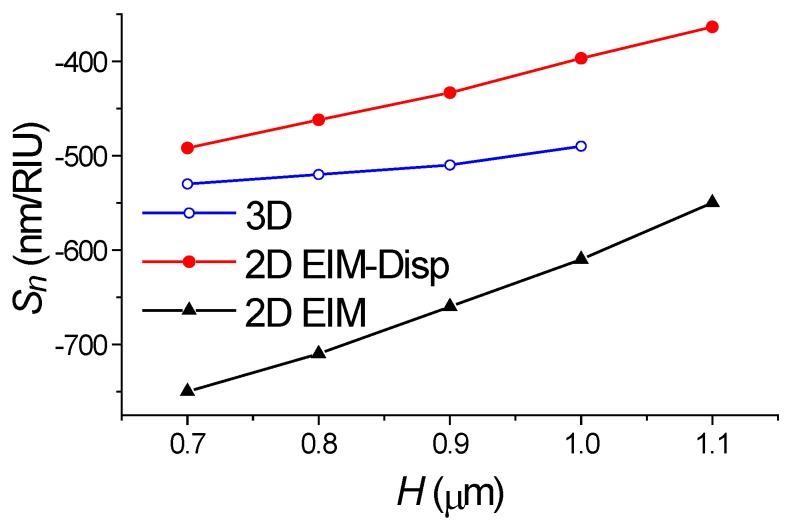
The dependence of the slope change *S_n_ =* ∂*λ/∂n* on the index of surrounding medium (water). Calculation by finite difference time domain (FDTD) method for 3D and 2D cases. 2D modeling is accomplished using the standard EIM and the dispersion compensation algorithm (EIM-Disp).

**Figure 6:**
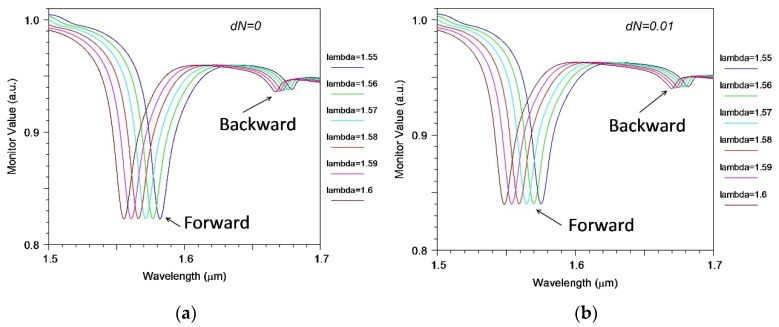
Transmission spectrum of silicon waveguide in the presence of a periodic segmented structure surrounded by water, calculated for different wavelengths of pulsed excitation and different small changes *dN* refractive index of the environment: (**a**) *dN* = 0.0; (**b**) *dN* = 0.01.

**Figure 7:**
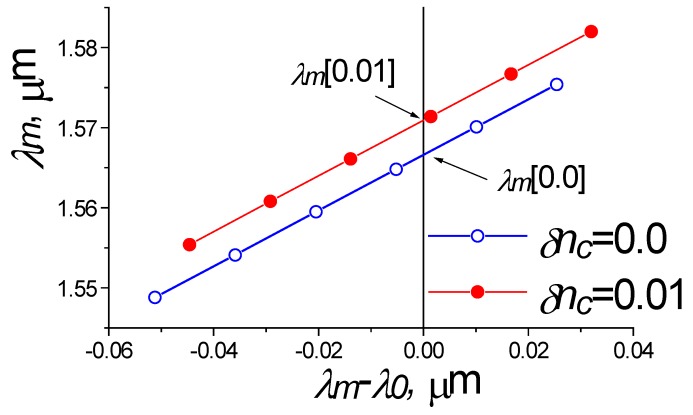
Dispersion compensation algorithm for two-dimensional simulation by FDTD method using effective index method (EIM).

This principal limitation of EIM has been also verified during the simulation of our segmented structures by 2D FDTD method. To illustrate the limitations of the conventional EIM, we calculated the sensitivity (see Figure 5) and spectral properties (see Figure 6 and Figure 7) of the same 128-segment waveguide structure by direct numerical simulation using 3D FDTD method and by 2D FDTD method in the EIM approximation.

Figure 6 shows the transmission spectrum of a silicon waveguide in the presence of a periodic segment structure surrounded by water, calculated by the 2D FDTD method in the EIM approximation for different wavelength of pulsed optical excitation. It is seen that due to waveguide dispersion (the notch wavelength at which the minimum transmission is observed) depends on the operating wavelength of the optical pulse. To assess the sensitivity of the sensor, calculations have been also performed for a small change (0.01) of the refractive index of the environment (see Figure 6b). Comparison of the data plots on Figure 6a and Figure 6b allows to determine the most important characteristic of the optical sensor, namely the dependence of the slope *S_n_ =* ∂*λ/∂n* of the variation of notch optical wavelength of the abnormal blocking on the change of the refractive index of the surrounding medium (water). 

Comparison of the exact calculation data by the 3D FDTD method with the results of 2D FDTD in the EIM approximation shows (see Figure 5) that the presence of the waveguide dispersion leads to 40% overestimation of sensitivity through the application of 2D FDTD plus EIM, which would seem to make calculations using 2D FDTD unsuitable for accurate analysis of such structures. 

Nevertheless, we found an original solution to this problem. As it was shown above, the presence of waveguide dispersion leads to the fact that the wavelengths of abnormal blocking *λ_m_,* found during the two-dimensional FDTD modeling, depends on the wavelength *λ*_0_, at which the impulse excitation and analysis of the spectrum of the waveguide structure is carried out. However, for the case *λ_m_ =* *λ*_0_ this value is exactly equal to the desired one. In other words, for the linear interpolation one can got the relation:

*λ_m_ = a_m_ + b_m_*(*λ_m_* − *λ*_0_) (5)

where *a_m_* and *b_m_* are the fitting constants of the linear approximation, in which *a_m_* is equal to the exact value of the desired notch wavelength in the investigated structure. It can be found from the approximation (2) or from graphic presentation. Therefore, by constructing the dependence *λ_m_* of the difference *λ_m_* − *λ*_0_ (see Figure 7) and having determined the values *λ_m_* at the zero coordinate, it is possible to find the wavelengths of the abnormal blocking for different values of the environment perturbation of the refractive index and, thereby, to determine the correct value *S_n_*, which differs slightly (see Figure 5) from the results of cumbersome 3D FDTD modeling (which usually requires at least 7 h on an eight-core personal computer).

### 2.4. The Refractometric Sensitivity of the Silicon Wire with the Segmented Grating

**Figure 8:**
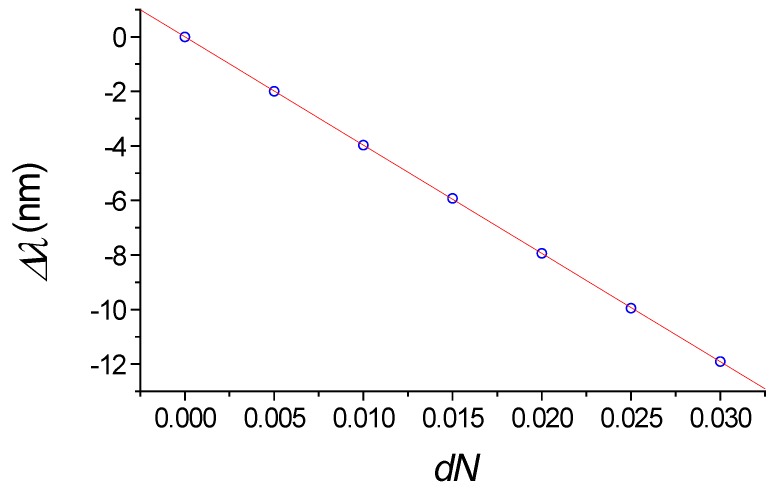
Dependence of drop wavelength shift as a function of index change *dN* in the water. *W* = *H* =1.0 µm. The simulation by 2D FDTD.

**Figure 9:**
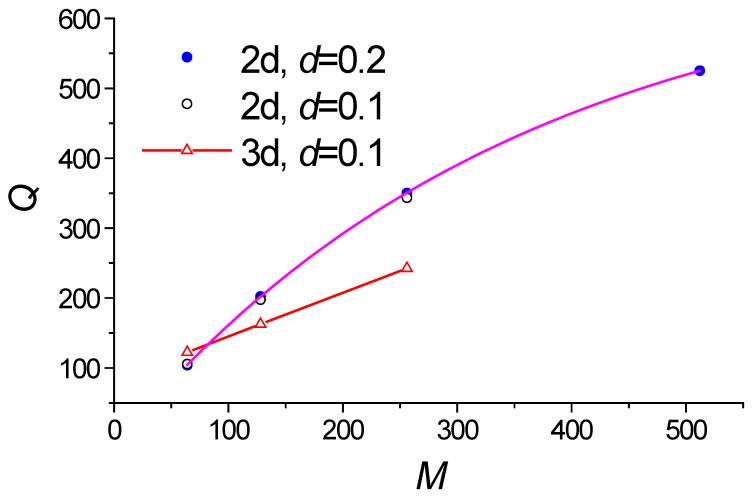
Dependence of the quality factor *Q* on the number of grating strips *M*. *W* = *H* =1.0 µm. The simulation by 2D FDTD and 3D FDTD.

This algorithm makes it possible to quickly and efficiently analyze and optimize the parameters of these segmented periodic structures and sensors based on them, using a combination of both effective index method and 2D FDTD modeling. This sensor element provides linear dependence of the wavelength shift on the environment index change. The results of 2D FDTD simulation for the structure with *W = H* =1.0 µm provides the refractometric sensitivity *S_n_* = 397.2 ± 0.7 nm/RIU, which is obtained from liner slop of the dependences ∆*λ* on *dN* shown on Figure 8. The quality factor *Q =* *λ_m_/*∆*λ*, where ∆*λ* is the full width half maximum (FWHM) is strongly depend on the on the number of grating strips *M* (see Figure 9). But its value is limited by the loss of the leaky wave. In our case, the maximum possible *Q* = 580 which is determined from exponential approximation of the data shown on Figure 9. Normally, the pick position could be measured with the accuracy about 1/15 of the FWHM. Thus this sensor can provide the moderate detector limit about 4 × 10^−4^.

The homogeneous sensitivity of the proposed sensor has a high value (around 500 nm/RIU as it is shown by 3D FDTD simulations) which is 7 times larger the typical value 70 nm/RIU for a normal-waveguide-based ring resonator [22] and is much higher than the experimental value 298 nm/RIU for a slot-waveguide-based ring resonator in Silicon on Insulator [16]. The experimental value for a slot-waveguide-based sensor is smaller than the theoretical estimated value 348 nm/RIU as the thin 100 nm slot region could not be completely filled with liquid. For a large period (1.3 µm) segmented grating this effect is negligible and the real sensitivity will be closer to the results of the modeling. In general, the homogeneous sensitivity of the proposed sensor is of the same order as in a subwavelength grating (SWG) sensor based on the ring resonator [17,18], but the last needs more robust technology (similar to slot-waveguide-based sensor) in order to manufacture thin tranches (around 100 nm) of the SWG waveguides and besides it has a similar problem of sensor sensitivity degradation due to incompletely filling with liquid. 

The problem of liquid filling of the thin slots makes possible the reduction in accuracy and in time delay on measuring the dynamic variation on index change in the structure environment. It seems that the smaller slot width the more difficult for the liquid filling with new index perturbation to replace an already filled slot by the liquid with the previous (in time) the index perturbation. Thus, the new sensor design which provides the record homogeneous sensitivity and four times larger slot gap could be interesting for the sensor applications. The limitation of this sensor is the rather small *Q* factor, thus it will be preferable in such kind of application where the moderate detection limit is not as principal as the high sensitivity, more effective grove filling by the liquid and the simpler technology manufacturing.

### 2.5. The Physical Nature of the Virtual Leaky Wave in Segmented Grating Structure in the Vicinity of the Silicon Wire Waveguide

We describe the effect of the abnormal blocking that provides the strong censoring effect by the interaction of the guided optical wave with the virtual leaky mode and is supported by the silicon wire with the segmented grating. The physical nature of the “virtual leaky mode waveguide” in this segmented structure is rather complicated and we have done a set of numerical modeling experiments by the 2D FDTD method for better understanding. 

**Figure 10:**
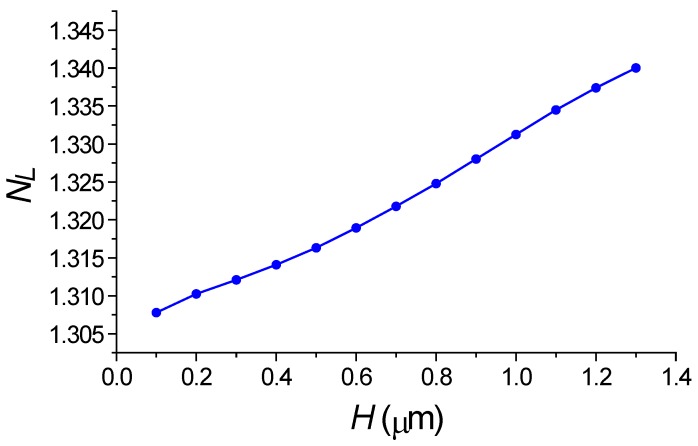
Dependence on the segmented width H of the mode index of the virtual leaky wave. *M* = 256, *W* = 1.0 µm. Simulation made by 2D FDTD.

**Figure 11:**
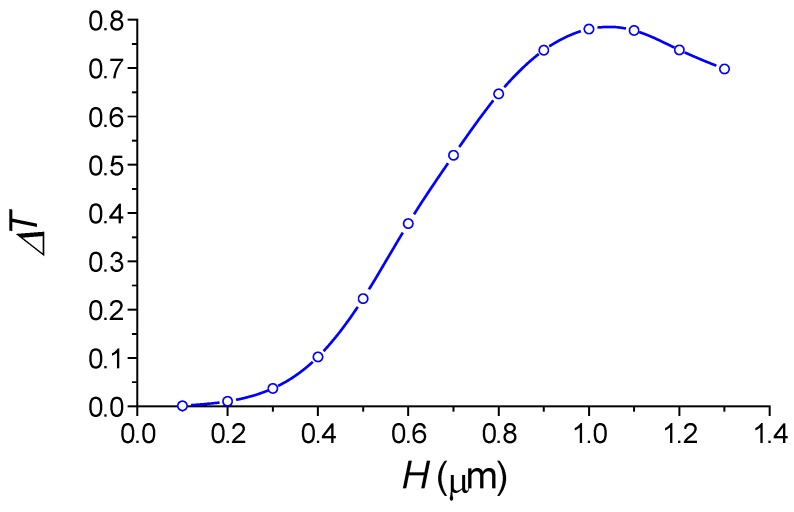
Dependence on the segmented width *H* of the transmitted power in silicon waveguide at the blocking wavelength. *M* = 256, *W* = 1.0 µm, *h* = 0.25 µm, *w* = 0.45 µm, *d* = 0.1 µm. Simulation made by 2D FDTD.

**Figure 12:**
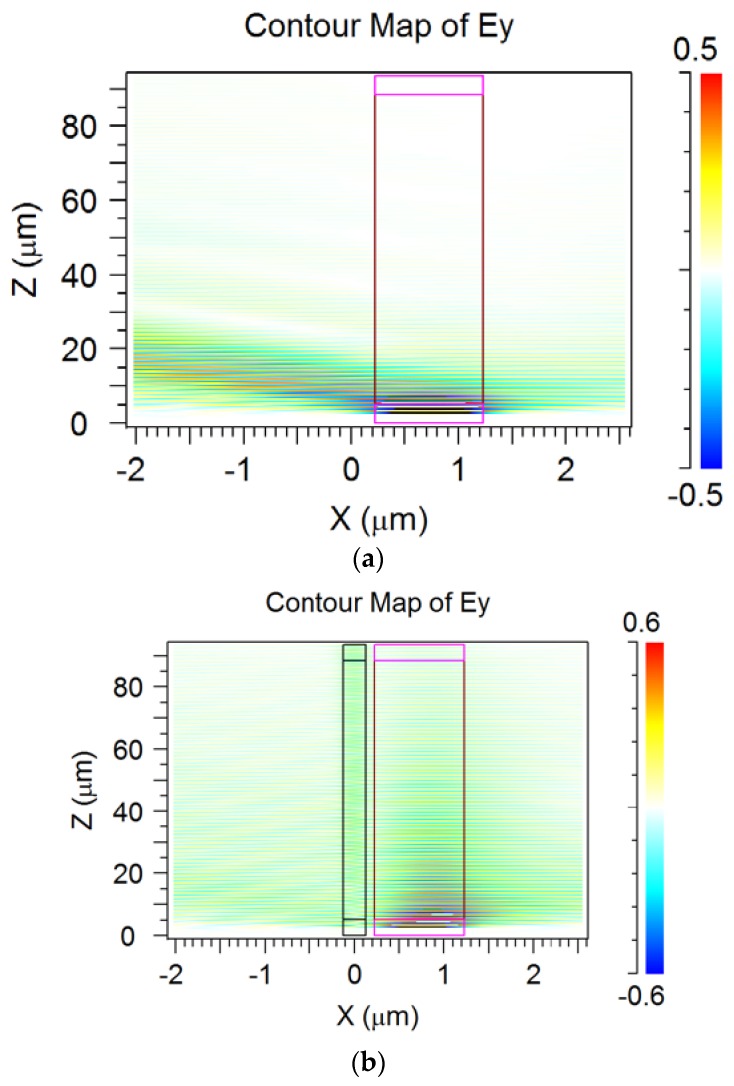
Counter plot of electric field Ey for the Gaussian optical beam launched into polymer strip connected with segmented grating. (**a**) Without silicon waveguide; (**b**) with silicon waveguide on a distance d from the grating. *M* = 128, *W* = 1.0 µm, *h* = 0.25 µm, *w* = 0.45 µm, *d* = 0.1 µm. Simulation made by 2D FDTD. Asymmetric radiation to the environment due to SiO_2_ on the left and water on the right from the grating structure.

**Figure 13:**
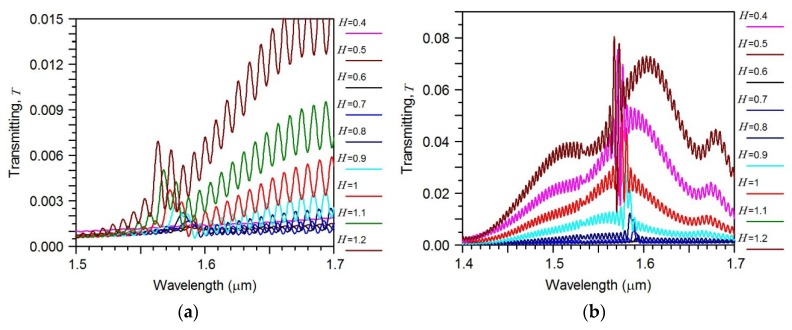
Transmitting power as a function of optical wavelength of the Gaussian optical beam launched into polymer strip connected with segmented grating of different height *H*. (**a**) *M* = 128; (**b**) *M* = 256, *W* = 1.0 µm, *h* = 0.25 µm, *w* = 0.45 µm, *d* = 0.1 µm. Simulation made by 2D FDTD.

At first, we examined the transmission spectrum of the silicon waveguide in the presence of the periodic segmented structure surrounded by water for different grating height *H.* We find the wavelength of the drop wavelength and relative transmitted power of the fundamental mode in the silicon wire. By taking into account the relation (2), we determine the effective index *N_L_* of the virtual leaky mode that is slowly increased with *H* (see Figure 10). Its value is close to the environment water, which results in the high sensitivity to the index change. The dropping efficiency grows with *H* and gets the maximum at *H* = 1 µm (see Figure 11). It is evident that all these are results of the presence of the segmented grating structure near the silicon wire. It is interesting that this segmented grating with the large period of 1.3 µm but is placed alone, never supports the guided wave propagation. For the case of launching the optical beam into this grating, all the power will be scattering into the balk wave as shown in Figure 12. Thus, no energy will pass to the grating end. The situation is drastically changed due the presence of the silicon wire in the grating vicinity (see Figure 13). The pair of silicon waveguide and segmented grating, constructs the structure which supports transmitting power along the waveguide axis as the virtual leaky wave having the small effective index (see Figure 11), and that is very sensitive to the grating environment (see Figure 5). 

Note that optimal grating height *H* = 1 µm is below the cutoff height 1.3 µm to support the fundamental mode in the polymer waveguide having the same index, width and height and the same environment. But, if the optical beam is launched into the segmented grating, it couples with the silicon wire and can propagate along the structure having a power exchange with it.

**Figure 14:**
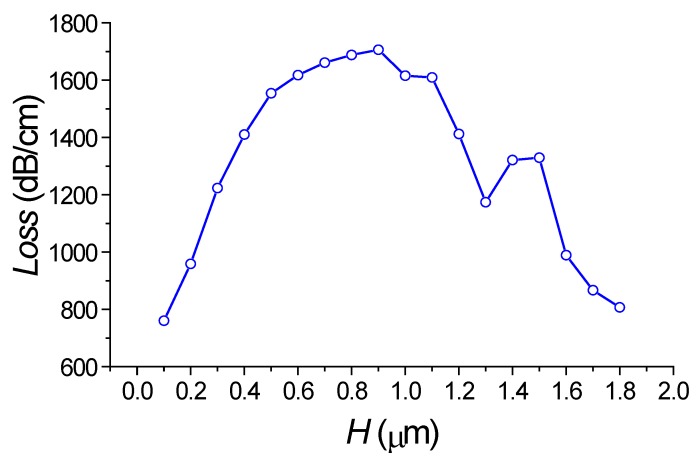
Dependence of the propagation loss in segmented grating obtained as the transmitting power of the Gaussian optical beam launched into polymer strip connected with segmented grating of different height *H*. *M* = 256, *M* = 128, *W* = 1.0 µm, *h* = 0.25 µm, *w* = 0.45 µm, *d* = 0.1 µm. The simulation by 2D FDTD.

The longer structure—the more power is concentrated in the silicon wire and thus part of the total power is concentrated in the grating area. This causes the ripples in the wavelength response of the power transmitted through the segmented grating to occur, depending on the number of segments (see Figure 12). Nevertheless, the total propagation optical loss in the segmented grating placed near the silicon wire is extremely high (see Figure 13 and Figure 14). In spite of this high optical loss, the guided to leaky wave transition has a resonant nature and provides a rather large quality factor *Q* that increases with the number of segments (see Figure 9).

**Figure 15:**
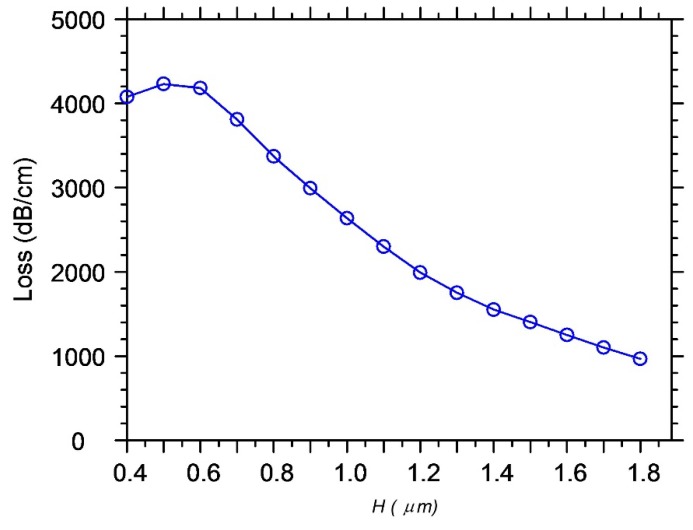
Dependence of the propagation loss in the segmented grating placed near the silicon semi space as a function of the different grating height *H*. It is obtained as the transmitting power of the Gaussian optical beam launched into polymer strip connected with segmented grating. *M* = 128, *W* = 1.0 µm, *h* = 0.25 µm, *w* = 0.45 µm, *d* = 0.1 µm. Simulation made by 2D FDTD.

The last set of simulations shows that the nature of virtual leaky mode is not typical. It exists due to the constructive interference of multiple reflections from the bottom silicon waveguide of radiated power scattered by the grating (compare Figure 12a,b) and it has an extremely large optical loss >700 dB/cm (see Figure 14). This virtual leaky mode exchanges power with the silicon waveguide that makes complicated to analyze the propagation loss of the “stand alone” virtual leaky mode. For its study we have replaced the silicon wire by the silicon semi space. This structure also supports the virtual leaky mode which is now “stand alone”. Thus, its propagation features have a slowly dependence on optical wavelength or the structure dimensions. The propagation loss of this virtual leaky mode is shown in Figure 15. This jointly with Figure 10 completely describe the optical properties of the virtual leaky mode which is supported by the periodic segmented structure placed in the vicinity of the silicon wire waveguide.

### 2.6. The Notch Filter Effect in the Silicon Wire Coupled with the Long Grating Structure

By increasing the number of segmented strips in the periodic grating, it is possible to provide the full suppression of the fundamental guided wave, which propagates along the silicon wire. It is complicated or impossible to study such long structures by the FDTD method (with reasonable limitations on memory and calculation time). Thus, the large-dimensional structures were additionally analyzed by MATLAB (MathWorks, Natick, NJ, USA) [23] software programs through the semianalytical matrix algorithm named the method of lines (MoL) [24,25,26]. It allows one to accurately describe the transmitting and reflection of the optical wave in structures with an arbitrary number of periodic segments (1000 or more) [12], which is beyond the capability of the 2D FDTD method.

**Figure 16:**
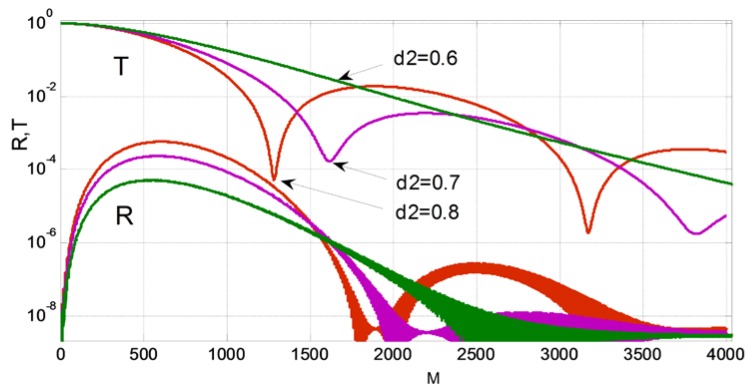
The effect of abnormal blocking for the forward diffraction. The dependence of the transmitting and reflection power of the fundamental mode of silicon wire on the number of segments (periods). The optimal number of grating periods *M* = 1280 and 3170 at *d*1 *= d*2 = 0.8 µm, (*Dd* = 0.0 µm, vertical boundaries) and *M* = 1615 and 3820 at *d*2 = 0.7 µm (*Dd* = 0.1 µm, weak boundary slope) and at *d*2 = 0.6 µm (*Dd* = 0.2 µm, strong boundary slope). The optimal wavelength at the barrier: *λ*_0_ = 1.55345 µm, 1.55580 µm, 1.55810 µm, respectively. Silicon oxide thickness *d* = 0.3 µm, refractive index of polymer segments *n_p_* = 1.521, height *H* = 1.0 µm, grating period *d*1 *+ d*0 = 1.6 µm. Analysis made using 2D MoL plus EIM.

**Figure 17:**
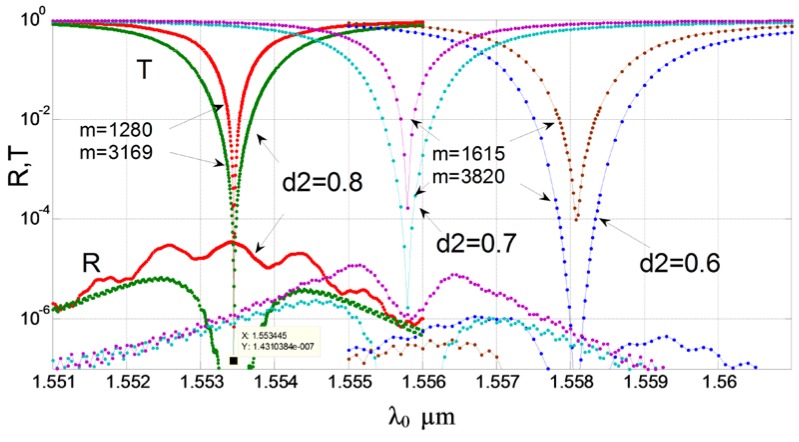
The effect of abnormal blocking for the forward diffraction. The dependence of the transmitting and reflection power of the fundamental mode of the silicon wire on the wavelength for different optimal number of segments (*M* = 1280, 3169, 1615 and 3820). Each slope of the walls corresponds to its optimal number of segments and its abnormal blocking wavelength (with *d*2 = 0.8 µm, vertical boundaries). Suppression of the transmitting wave is at level of −40 to −60 dB. Analysis made using 2D MoL plus EIM.

**Figure 18:**
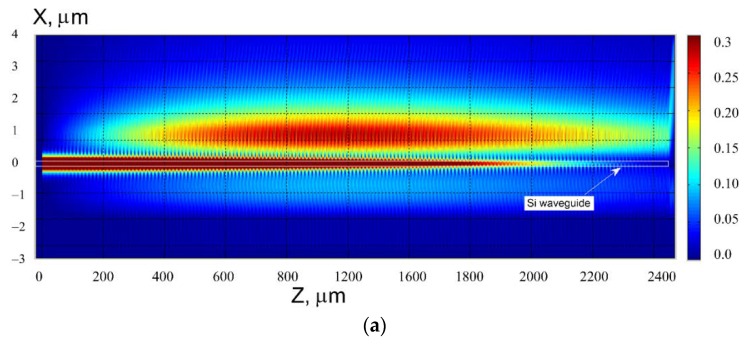

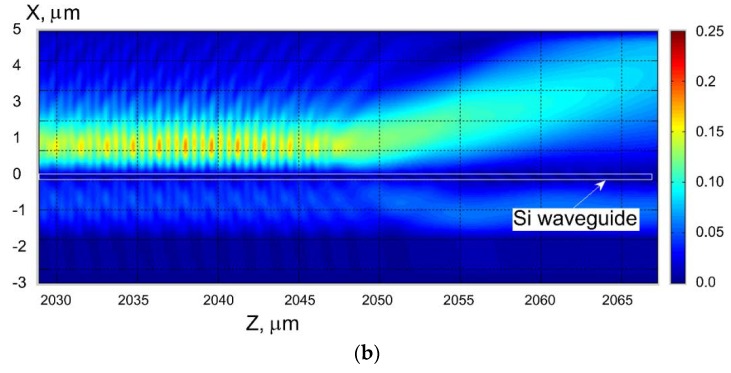
Illustration of the abnormal blocking effect for forward diffraction. (**a**) Total structure view; (**b**) enlarged portion of the right part of the structure. The segmented grating period is 1.6 µm, the number of polymer segments *M* = 1280, the evanescent gap *d* = 0.3 µm. The case of vertical segment boundaries (*d*1 *= d*2 = 0.8 µm). The thickness of the silicon wire is 0.22 µm. Analysis made using 2D MoL plus EIM.

**Figure 19:**
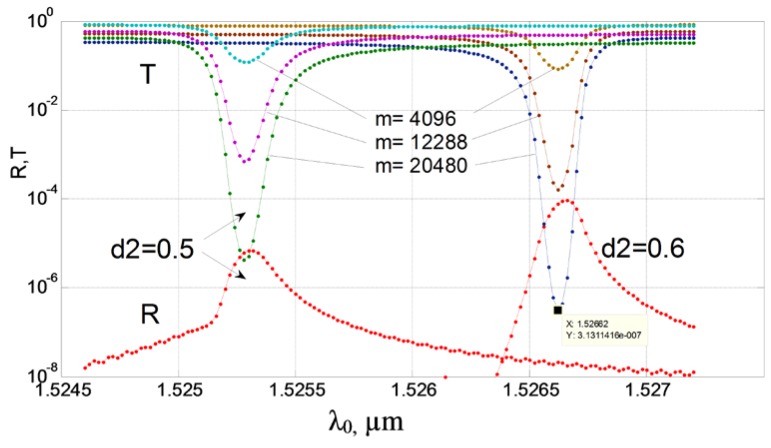
The effect of abnormal blocking for the backward diffraction. The dependence of the transmission power and reflection of the fundamental mode of the silicon wire on the wavelength for different number of segments (*M* = 4096, 12,288 and 20,480). Two operating wavelengths *λ*_0_ = 1.52528 µm and 1.52662 µm for the inclined segment boundary (*d*2 = 0.5 µm) and the vertical segment boundary (*d*2 *= d*1 = 0.6 µm), respectively. The evanescent gap *d* = 0.25 µm, the thickness of the polymer *H* = 1.25 µm, 1.2 µm period. Analysis made using 2D MoL plus EIM.

**Figure 20:**
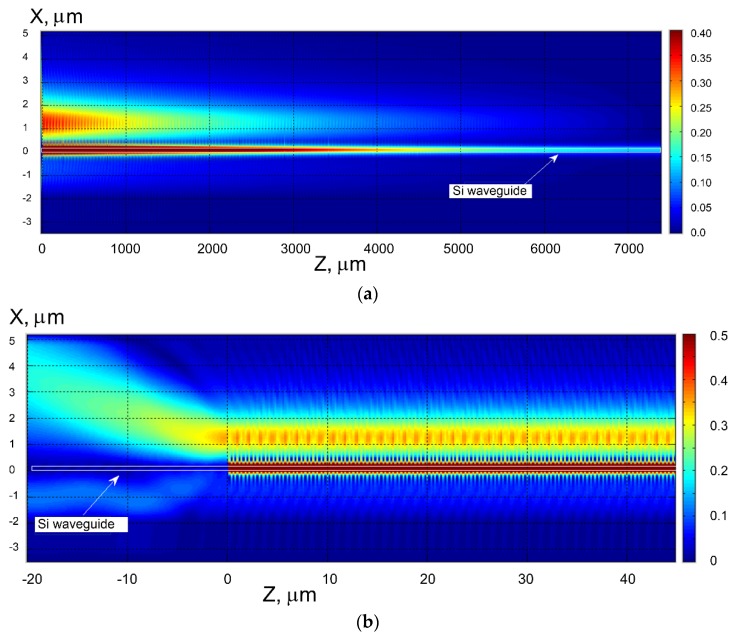
Illustration of the abnormal blocking effect for back diffraction. (**a**) Total structure view; (**b**) enlarged portion of the right part of the structure. Here the segmented grating period is 1.2 µm, the number of polymer segments *M* = 6144, the evanescent gap *d* = 0.25 µm. The thickness of the silicon wire is 0.22 µm. The case of vertical segment boundaries (*d*1 *= d*2 = 0.6 µm). Analysis made using 2D MoL plus EIM.

The results of calculation by the method of lines of optimal structures, in which the effect of abnormal blocking is observed, are given in Figure 16, Figure 17, Figure 18, Figure 19 and Figure 20 for the cases of diffraction into the leaky wave of the segment structure for forward and backward processes, respectively. We consider segments with both vertical and inclined boundaries. In general, the efficiency of the abnormal blocking is determined by the optical index contrast of the segments and the environment, the size, period and slope of the segment boundaries, as well as the value of the weak coupling gap. 

The number of segments must also be the optimal number for every set of parameters. For example, for the forward diffraction from guided to virtual leaky wave (see Figure 16) taking place for the first grating order (*p*), we observe the almost total suppression (more than −40 dB) of the input guided wave on the vertical etched segmented structure with the 1280 grating periods. With the larger number of periods, we see the reversal process of power transmitting from leaky to guided wave but, due to the coupling to the radiation modes, the residual power in the fundamental guide mode is very small and strongly decreases for the next optimum number of grating periods *M* = 3170. One can see that the back reflected guided wave is also negligible. 

The spectral properties of this structure for the optimal number of segments (to provide the maximum guided wave blocking) is shown in Figure 17. The effect of abnormal blocking can be seen with a small line width of the resonant coupling of guided to virtual leaky mode, but the smaller line width occurs for the smaller number of segments. The reflection power of the guided mode is negligible for the wide spectral range we have examined. It can be noted that the slop wall of the segments does not produce any significant change in the wavelength response, but the shift in the drop wavelength and in line width are noticeable. The next Figure 18 illustrates the abnormal blocking effect for forward diffraction by showing the cross distribution of the electric field. As the optical wave propagates from left to the right in the silicon wire, the energy is completely concentrated in the segment strips (upper red spot), and then re-emitted into the free space. We see in the Si-wire a complete depletion of energy to occur (abnormal blocking), while any reflected mode cannot be revealed (no field in the left edge of the figure). For the better illustration of this phenomena, the Figure 18b shows the enlarged pattern on the right part of the structure. We see the edge energy emission (which is not radiated before in the propagation of the leaky mode) from the segment structure located above the silicon wire. The last segment is located at *Z* = 2048 µm. It is clearly seen that for the optimal number of polymer segments (*M* = 1280) it does not remain any energy in the Si-waveguide.

The similar effect takes place for the backward diffraction of the 3rd diffraction order by the segmented grating (see Figure 19). It is interesting that for the back-reflection process of the abnormal blocking, the energy in the back reflected fundamental mode in the silicon waveguide is very small (below −40 dB). This fact is illustrated by the cross section of the electric field shown on Figure 20. As the wave propagates to the right in the silicon wire, the energy is coupled to the location of segmental structure and wrapped back (see top spot on the left). If we continue the structure on the right beyond the border of the figure, the field in the waveguide approaches to zero, too. At the same time, any reflected mode cannot be seen in the Si waveguide. In the enlarged pattern (see Figure 20b), it is better seen the edge radiation of energy to the left from the segment structure located above the silicon wire. The left corner segment is located at *Z* = 0. It is clearly seen that when *Z* <0 we do not have any reflected mode (see Figure 20, reflection coefficient *R*~10^−4^) in the silicon wire, and all the energy is re-radiated by polymer segments located above the silicon waveguide. One must note that the back reflected process should be suitable to construct notch filters but the wavelength sensitivity to the index change in the environment is several times smaller than that for the forward diffraction. Thus, the last case is more suitable for implementation in optical sensing (as shown in Section 2.4).

## 3. Conclusions

In this work, it is shown that due to the collective scattering by the multiple weak coupled periodic strips, almost a complete blocking propagation of the TE_0_ mode both in forward and reverse directions can be achieved. This new effect is a result of the resonant fundamental guided mode interaction in the silicon wire with the virtual leaky mode, which is localized in the fully etched polymer grating with a period of 1.3 µm and placed about 100–400 nm above the silicon wire through a thin silicon oxide layer. In this case, the blocking optical signal is transmitted into the leaky segmented waveguide and then further scattered into the waveguide environment as a bulk wave. Differently from high-order Bragg gratings reflection (that takes place in the structure, too), the scattering into the leaky mode by evanescent coupling between the silicon layer and the periodic strips is very strong and does not lead to any back-reflection of the guided mode in the silicon wire. On the contrary, to traditional grating-assisted coupling for guided to radiation mode, the effect of the abnormal blocking provides a small line width at which the guided wave is transmitted to radiation mode. Besides, the effective index of the leaky mode due to presence of the segmented structure is very close to the external environment [12], and thus the guided to leaky wave interaction is very promising for sensing applications. This effect of abnormal strong guided wave blocking by multiple low index strips with a weak coupling could be also used for the construction of novel types of notch filters with negligible back-reflection.

We have performed numerical simulations by both the FDTD method and MoL. These simulations showed that the segmented periodic structures, evanescently coupled to the underlying silicon wire, provides high homogeneous sensitivity *S_n_ = ∂λ/∂n* = 500 nm/RIU of the wavelength of the abnormal blocking to the change of the refractive index of the surrounding space (water, in our case) and can be used as optical sensors. The detector limit—about 4 × 10^−4^ of the sensor—is limited by the moderate *Q*-factor (580), which is limited by the optical loss of the leaky wave that is supported by the grating structure. The sensor sensitivity is significantly higher than that of a silicon-based sensor [22] and it is comparable in value with the best SWG grating and slot optical sensors [17]. However, the last structures require nanotechnology, as the fabrication of which both high-precision electron beam lithography and submicron etching are needed [22] to achieve narrow gaps (about 100 nm) in a slot or segmental waveguides structures. The characteristic gaps of the segment structure of the proposed optical sensing approach is four times larger, which makes it possible to produce them based on simplified and cheaper technologies, including the use of standard UV optical lithography and standard etching. The other advantage of proposed design is in the simpler liquid filling of the large (in 4 times magnification) slots in comparison with the conventional SWG and slot structures, which will increase the accuracy of dynamic measuring in an index change of the environment due the simpler slot being filled by the liquid.

## References

[1] Marcuse D. (1987). Directional couplers made of nonidentical asymmetric slabs. Part II: Grating-assisted couplers. J. Lightw. Technol..

[2] Zhang S.H., Tamir T. (1997). Grating-assisted couplers with broadband characteristics. Opt. Lett..

[3] Passaro V.M.N. (2000). Optimal design of grating-assisted directional couplers. J. Lightw. Technol..

[4] Taillaert D., Bienstman P., Baets R. (2004). Compact efficient broadband grating coupler for silicon-on-insulator waveguides. Opt. Lett..

[5] Doylend J.K., Knights A.P. (2006). Design and Simulation of an Integrated Fiber-to-Chip Coupler for Silicon-on-Insulator Waveguides. IEEE J. Sel. Top. Quantum Electron..

[6] Kwon M.-S., Shin S.-Y. (2004). Tunable Notch Filter Using a Thermooptic Long-Period Grating. J. Lightw. Technol..

[7] Helfert S.F., Pregla R. (1998). Efficient Analysis of Periodic Structures. J. Lightw. Technol..

[8] Alferness R.C., Koch T.L., Buhl L.L., Storz F., Heismann F., Martyak M.J.R. (1989). Grating-assisted InGaAsP/InP vertical codirectional coupler filter. Appl. Phys. Lett..

[9] Shi W., Wang X., Lin C., Yun H., Liu Y., Baehr-Jones T., Hochberg M., Jaeger N.A.F., Chrostowski L. (2013). Silicon photonic grating-assisted, contra-directional couplers. Opt. Express.

[10] Zhang Z., Genrich G., Keil N., Grote N. (2014). Widely tunable grating-assisted heterogeneous silicon nitride/polymer waveguide coupler. Opt. Lett..

[11] Tsarev A.V. (2009). New wide strip and grating loaded quasi-single-mode waveguide on SOI. Opt. Express.

[12] Kolosovskii E.A., Tsarev A.V. (2017). Abnormal blocking of a guided mode propagating in a silicon optical waveguide with periodic tunnel coupling. Quantum Electron..

[13] Roelkens G., Dumon P., Bogaerts W., Van Thourhout D., Baets R. (2005). Efficient silicon-on-insulator fiber coupler fabricate during 248-nm-deep UV lithography. IEEE Photonics Technol. Lett..

[14] Rsoft FullWave by SYNOPSYS Photonic Design Software. https://www.synopsys.com/optical-solutions/rsoft.html.

[15] Dell’Olio F., Passaro V.M.N. (2007). Optical sensing by optimized silicon slot waveguides. Opt. Express.

[16] Claes T., Molera J.G., De Vos K., Schacht E., Baets R., Bienstman P. (2009). Label-free biosensing with a slot-waveguide-based ring resonator in silicon on insulator. IEEE Photon. J..

[17] Schmidt S., Flueckiger J., Wu W., Grist S.M., Talebi Fard S., Donzella V., Khumwan P., Thompson E.R., Wang Q., Kulik P. Improving the performance of silicon photonic rings, disks, and Bragg gratings for use in label-free biosensing. Proceedings of the Biosensing and Nanomedicine VII.

[18] Flueckiger J., Schmidt S.V., Donzella Sherwali A., Ratner D.M., Chrostowski L., Cheung K.C. (2016). Sub-wavelength grating for enhanced ring resonator biosensor. Opt. Express.

[19] Chiang K.S. (1986). Dual effective-index method for the analysis of rectangular dielectric waveguides. Appl. Opt..

[20] Tsarev A. (2013). Modified effective index method to fit the phase and group index of 3D photonic wire waveguide. Opt. Lett..

[21] O’Faolain L., Tsarev A. (2014). Experimental demonstration of original optical filter based on multiply coupled waveguides. Opt. Lett..

[22] De Vos K., Bartolozzi I., Schacht E., Bienstman P., Baets R. (2007). Silicon-on-insulator microring resonator for sensitive and label-free biosensing. Opt. Express.

[23] MathWorks. www.mathworks.com.

[24] Rogge U., Pregla R. (1991). Method of lines for the analysis of strip-loaded optical waveguides. Opt. Soc. Am. B.

[25] Scarmozzino R., Gopinath A., Pregla R., Helfert S. (2000). Numerical techniques for modeling guided-wave photonic devices. IEEE J. Sel. Top. Quantum Electron..

[26] Jamid H.A., Akram M.N. (2002). Analysis of Deep Waveguide Gratings: An Efficient Cascading and Doubling Algorithm in the Method of Lines Framework. J. Lightw. Technol..

